# Management of Adult Obstructive Sleep Apnoea: Many Questions, Not Enough Answers!

**DOI:** 10.1111/jsr.70047

**Published:** 2025-03-24

**Authors:** Maria Stanczyk, Walter T. McNicholas, Dirk A. Pevernagie, Renata L. Riha, Silke Ryan

**Affiliations:** ^1^ Pulmonary and Sleep Disorders Unit St. Vincent's University Hospital Dublin Ireland; ^2^ School of Medicine University College Dublin Dublin Ireland; ^3^ Department of Respiratory Diseases AZ Delta Roeselare Belgium; ^4^ Department of Internal Medicine and Paediatrics, Faculty of Medicine and Health Sciences Ghent University Ghent Belgium; ^5^ Department of Sleep Medicine, Royal Infirmary of Edinburgh University of Edinburgh Edinburgh Scotland

**Keywords:** cardiometabolic complications, CPAP, obstructive sleep apnoea, phenotyping

## Abstract

Obstructive sleep apnoea (OSA) conveys a substantial global public burden due to its high prevalence and causative relationship with cardiometabolic diseases. The current diagnostic reliance on the apnoea/hypopnoea index (AHI) is insufficient to address the complex, multifaceted condition, and a revision of the standard criteria is urgently needed. Together with a better understanding of the clinical, pathophysiological, and sleep diagnostic phenotypic characteristics, this will pave the way to personalised, holistic treatment approaches.

## 1 Introduction

Obstructive sleep apnoea (OSA) constitutes a major global public health problem with a high and steadily rising prevalence, mainly attributed to its intimate relationship with obesity (Benjafield et al. 2019). The disorder occurs as a result of repetitive partial or complete occlusion of the upper airway (UA) leading, among others, to intermittent hypoxia (IH) and sleep fragmentation, which activate a broad spectrum of pathophysiological pathways that collectively contribute to the development of comorbidities, most notably cardiovascular (CV) and metabolic diseases (O'Donnell et al. 2021). These comorbidities, in turn, have a significant impact on healthcare utilisation, cost, and mortality (AASM 2016; Alakorkko et al. 2023; Labarca et al. 2023). Furthermore, untreated OSA represents a major risk factor for motor vehicle accidents, which have been reported to be 2–3 times more frequent in patients with OSA when compared to an equivalent general population (Bonsignore et al. 2021).

The most widely employed therapy of OSA over recent decades has been continuous positive airway pressure (CPAP). Although CPAP is highly effective in improving daytime sleepiness, quality of life, and other symptoms, its effect on cardiometabolic complications is uncertain. Moreover, the cumbersome nature of the device makes it unattractive to many subjects, and alternative treatment options are highly desirable. OSA represents a highly heterogenous condition, and the increasing understanding of different clinical and pathophysiological phenotypes favours a shift towards a personalised approach to the diagnosis and management of OSA.

This review will focus on important questions and ongoing controversies in this field and provide potential future directions.

## 2 Obstructive Sleep Apnoea—What Is It and What Matters for the Diagnosis?

First, it is important to establish that OSA has several alternative names that may or may not encapsulate the disorder under discussion. These include obstructive sleep apnoea syndrome (OSAS), sleep apnoea, sleep‐disordered breathing (SDB) and obstructive sleep apnoea/hypopnoea syndrome (OSAHS) (Riha 2021). Strictly speaking, one could argue that OSA should be reserved only for the pathological breathing changes at night and reserve the term OSAS (alternative OSAHS) if those lead to daytime symptoms such as excessive daytime sleepiness (EDS), fatigue, impairment in concentration, memory, or similar. The current diagnosis and severity grading of OSA (Table 1) are heavily dependent on the apnoea/hypopnoea index (AHI) (AASM 2023); however, this marker is clearly limited in its validity as a metric of the OSA‐related breathing disturbances (Pevernagie et al. 2020). Benjafield et al. estimated that, globally, 936 million people aged 30–69 years have at least an AHI ≥ 5/h and 425 million have an AHI ≥ 15/h (Benjafield et al. 2019) but the assumption that criterion on its own implies a disease status is not founded on medical evidence. The AHI correlates poorly with symptom burden such as EDS, neurocognitive performance, or quality of life (Arnardottir et al. 2016; Heinzer et al. 2015). Furthermore, as a predictor of cardiometabolic outcomes, the AHI is inferior to other sleep diagnostic markers such as those defining the severity of IH or autonomic dysfunction (Azarbarzin et al. 2019; Solelhac et al. 2023). It is also important to point out that the AHI demonstrates up to 20% variability from night to night and is in itself highly dependent on the technology used to measure it (Roeder et al. 2020). Hence, the mere use of the AHI as a predictor for disease status in the community probably leads to substantial overestimation of clinically relevant OSA.

**TABLE 1:** Diagnostic criteria for adult OSA (adapted from ICSD‐3‐TR (AASM 2023)): The diagnosis requires that the individual must fulfil criterion ((A and B), or criterion C) + D.

The presence of one or more of the following: – Complaints of sleepiness, fatigue, insomnia or other symptoms such as sleepiness whilst driving, impaired daytime concentration, decrements in memory etc. leading to impaired sleep‐related quality of life – The patient wakes with breath holding, gasping, or choking – Bed partner or others report habitual snoring, breathing interruptions, or both during patient's sleep
B PSG or HSAT demonstrates: – 5–14 predominantly obstructive respiratory events (apnoeas, hypopnoeas, or RERAs) per hour of sleep (PSG) or per hour of monitoring time (HSAT)
C PSG or HSAT demonstrates: – 15 or more predominantly obstructive respiratory events (apnoeas, hypopnoeas, or RERAs) per hour of sleep (PSG) or per hour of monitoring (HSAT)
D The symptoms are not better explained by an another organic sleep disorder, medical disorder, medication or substance abuse.

Abbreviations: HSAT (home sleep apnoea test); PSG = polysomnography; RERA: respiratory effort‐related arousal.

The shortcomings of the AHI‐based approach highlight the importance of including more clinical elements in the diagnostic context. EDS is considered the most debilitating symptom of OSA and, importantly, contributes independently to cardiovascular outcomes (Mazzotti et al. 2019). However, the symptom is rather unspecific and is experienced in a number of somatic, psychiatric, and primary sleep disorders, but also occurs physiologically in the absence of sufficiently restorative sleep periods (Brown and Makker 2020). Studies have often dichotomized subjects into sleepy and non‐sleepy based on a very arbitrary cut‐off value of the Epworth Sleepiness Scale (ESS) of 10, which has poor sensitivity and specificity, and the ESS in itself has numerous other limitations and is widely open to reporting bias (Chervin and Aldrich 1999). Furthermore, subjects are often accustomed to their symptoms and only realise the impact of their OSA on their quality of life after treatment initiation or may under‐report their level of sleepiness, potentially motivated by concerns in relation to a compromise in their professional activities or driving licensing (Leclerc et al. 2014; Strohl et al. 2013). In addition, although EDS is the classical daytime symptom of OSA, there is substantial heterogeneity in presentation, and especially women often report unspecific symptoms such as insomnia, irritability, fatigue, or nightmares (Basoglu and Tasbakan 2018). The detailed contribution of such symptom burden to OSA outcomes remains largely unexplored. Despite those considerations, the often observed discrepancy between the severity of sleep‐disordered breathing and symptom burden implies the involvement of additional factors that are probably rooted in genetic contributions determining the individual's susceptibility to the harmful systemic effects of respiratory disturbances during sleep (Pevernagie 2021). This aspect represents a promising but yet underexplored area of research.

Thus, the future of the diagnosis of OSA requires a better understanding of the susceptibility to adverse consequences in addition to adequate phenotyping incorporating markers of clinical and sleep diagnostic characteristics. Having a holistic and solid definition allows us to explore the pathophysiological underpinnings of the disease process, to develop biomarkers if possible, and to tailor treatments appropriately that lead to the best health outcomes. Furthermore, it will facilitate a better understanding of the true epidemiology of the condition, which is essential in the planning of appropriate global healthcare resources.

## 3 Sleep Diagnostics—What Is Needed?

As outlined above, the diagnosis of OSA needs to incorporate the clinical symptomatology and hence, a detailed longitudinal sleep/wake history and physical examination are critical in the evaluation of a person with suspected OSA. However, although the most appropriate diagnostic markers are still to be defined, the demonstration of sleep‐disordered breathing is essential. The gold standard for overnight monitoring is full polysomnography (PSG), which provides detailed information on sleep staging, respiratory and gas exchange abnormalities, in addition to a range of other variables including body position, heart rate and rhythm, and muscle tone and contraction (Berry et al. 2012). However, these studies are resource intensive since they generally require the facilities of a full sleep laboratory and a trained technician. Thus, the prevalence figures for OSA make it necessary to consider other simplified approaches to diagnosis. A variety of limited diagnostic systems are available. Polygraphy (PG) denotes a procedure for monitoring respiratory sleep disorders, whereby the recordings of neurophysiological signals determining sleep are omitted. It typically includes the measurement of oronasal airflow, chest wall and abdominal effort, electrocardiogram, and oxygen saturation. The potential advantages of these systems are that they are cheaper, less labour and time‐intensive, technically less challenging and thus, are often used for domiciliary testing (Masa et al. 2013). The main disadvantage is that the lack of sleep recording leads to uncertainty when deciding if respiratory events, particularly periods of normal respiration, occur during wakefulness or sleep. Due to the fact that even abbreviated methods for detecting sleep‐related breathing disorders are time‐consuming in their application, scoring and interpretation, there has been a flurry of consumer‐based devices developed to detect breathing pauses in sleep. None of those have been validated for general use in the diagnostic pathways of OSA to date but will likely increasingly serve as screening tools in the future (Pires et al. 2023). New computational paradigms and machine learning tools offer the opportunity to reduce the manual workload of diagnostic testing and investigate the potential for novel signal analysis techniques that may revolutionise the approach to OSA investigation and classification. These developments represent the major objectives of the ongoing EU Horizon 2020‐funded Sleep Revolution project (Arnardottir et al. 2022).

## 4 Pathophysiology of OSA—The Upper Airway and What Else?

**FIGURE 1:**
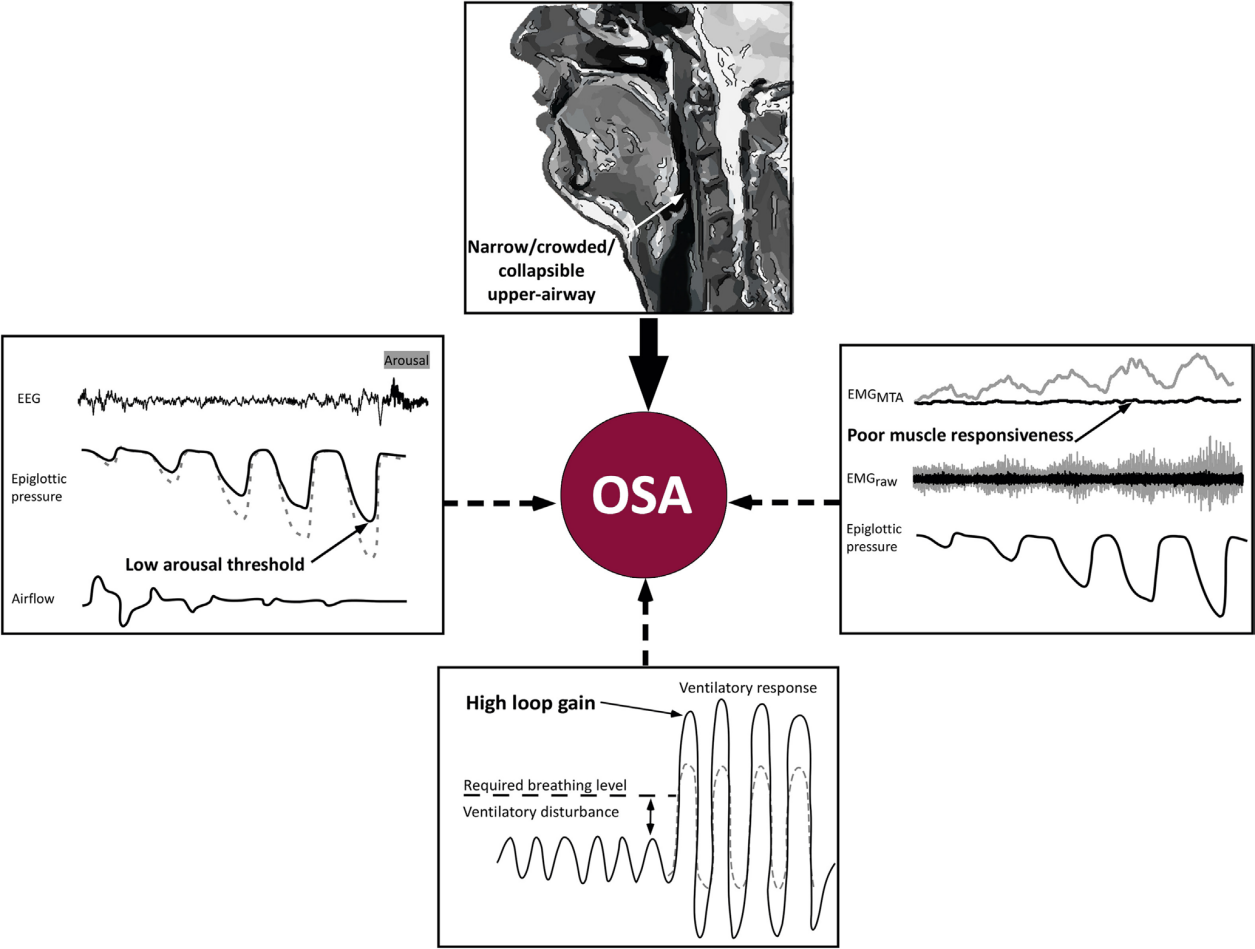
Schematic diagram of the pathophysiology of obstructive sleep apnoea incorporating anatomical and non‐anatomical factors (reproduced from Carberry et al. 2018). Figure is reproduced and requires permission.

As stated above, one reason for defining a disease such as OSA properly lies in determining the etiopathology correctly which in turn informs management of the disorder. While the core pathophysiology of OSA relates to an increased collapsibility of the pharyngeal airway, the underlying mechanisms are complex and multifactorial, representing an interaction between anatomical and non‐anatomical factors that together constitute the endotype of OSA in the individual patient (Carberry et al. 2018; Osman et al. 2018) (Figure 1). The key anatomical factor in the pathophysiology of OSA is a narrowed UA. Most OSA subjects have narrowing at the oropharyngeal level, which can be clinically graded by the Mallampati score (Yu and Rosen 2020). Genetic and hereditary factors are increasingly recognised as important in this structural narrowing (Chi et al. 2014). Such factors may reflect a nonspecific reduction in UA calibre, or specific defects in the bony and maxillofacial structures in the lower face and neck such as micrognathia or retrognathia, which result in posterior positioning of the tongue, thus predisposing to UA obstruction (McNicholas 2008a; Neelapu et al. 2017). Obesity is also an important contributor to UA narrowing, principally by deposition of neck fat encircling the UA (Deegan and McNicholas 1995), and other relevant anatomical factors include reversible nasal obstruction as occurs in rhinitis (McNicholas 2008b), and gravitational forces associated with the supine sleeping position (Yildirim et al. 1991).

In recent years, it has been increasingly recognised that non‐anatomical factors play potentially important roles in the pathophysiology of OSA, with about one third of subjects reflecting at least one of these traits. One of those factors is a decreased responsiveness of the pharyngeal dilator muscles, particularly the genioglossus muscle. Those must contract more forcefully to maintain a patent airway in the setting of UA narrowing, which is a particular challenge in sleep, as the activity of these muscles diminishes more in OSA patients than normal subjects during sleep, albeit from a higher baseline awake level (Carberry et al. 2016; Mezzanotte et al. 1996). Further potentially contributing factors include a high loop gain, reflecting a high sensitivity of the feedback loop modifying the ventilatory response to a respiratory disturbance (Jordan et al. 2005), and a low arousal threshold resulting in a high propensity to arouse from sleep (Eckert and Younes 2014). Both contribute to repetitive obstructive apnoea's through a vicious loop of ventilatory overshoot, hypocapnia, and subsequent reduction in ventilatory drive.

It is estimated that hereditary factors affecting the distribution of obesity, craniofacial features, and ventilatory control account for 40% of the variance in the occurrence of OSA in the population, with the rest being attributable to acquired factors (Redline and Tishler 2000). Notably, multiple pathophysiological traits occur in about 30% of OSA subjects (Eckert et al. 2013).

## 5 Cardiometabolic Comorbidities in OSA—What Is the Target?

Besides the high prevalence, the burden of OSA is primarily attributed to its frequent association with cardiovascular (CV) and metabolic comorbidities leading to considerable morbidity, mortality, health care utilisation, and cost (AASM 2016; Alakorkko et al. 2023; Javaheri et al. 2017; Kent et al. 2015; O'Donnell et al. 2021; Ronald et al. 1999). The benefit of CPAP therapy as the treatment of choice for the majority of OSA subjects on cardiometabolic disease processes remains uncertain, and, albeit impacted by important methodological limitations, secondary prevention trials have failed to demonstrate a significant modifying effect on CV outcomes (McEvoy et al. 2016; Peker et al. 2016; Sanchez‐de‐la‐Torre et al. 2020). CPAP therapy alone is also insufficient to improve metabolic functions (Chirinos et al. 2014; Jullian‐Desayes et al. 2015; Labarca et al. 2018), thus effective treatment strategies are urgently required.

A greater understanding of the detailed mechanisms underlying cardiometabolic disease processes in OSA will likely identify potential therapeutic targets, but despite significant efforts being made, the pathophysiology remains incompletely understood. However, there is extensive evidence that intermittent hypoxia (IH), the hallmark feature of the condition, plays a pivotal role. Notably, IH itself is considered a ‘double‐edged sword’ and short exposures to mild IH may lead to adaptive responses through preconditioning effects and hence may be cardioprotective for subjects with mild OSA (Almendros et al. 2014; Panza et al. 2022). However, the IH pattern typically seen in moderate to severe OSA, with frequent short cycles of IH with deeper desaturations, and prolonged exposures, leads to numerous deleterious responses. In support of a critical role of such IH in cardiometabolic processes, various epidemiological studies have identified the superiority of markers defining the severity of IH, such as hypoxic burden, in the prediction of adverse outcomes over the traditional AHI, which predominantly reflects airflow limitations (Azarbarzin et al. 2019; Pinilla et al. 2023). However, most of our evidence of the deleterious role of IH has arisen from experimental studies using cell culture, animal, or human models. Of particular relevance has been the rodent model of IH, and a recent extensive systematic review and meta‐analysis comprising 125 studies, adjusted for the heterogeneity of models with respect to severity of desaturations and duration of exposure, concluded that IH leads to vascular remodelling in a dose‐dependent fashion and acceleration of atherosclerotic plaque formation (Harki et al. 2022). In addition, several studies have also demonstrated adverse effects of IH on insulin sensitivity and other metabolic functions independent of the presence of obesity (Murphy et al. 2017; Thomas et al. 2017). IH likely mediates its adverse effects through a multitude of intermediate pathophysiological pathways including, among others, sympathetic activation, inflammation, and oxidative stress (Fletcher et al. 1992; Ryan 2018; Ryan et al. 2005; Ryan et al. 2009).

**FIGURE 2:**
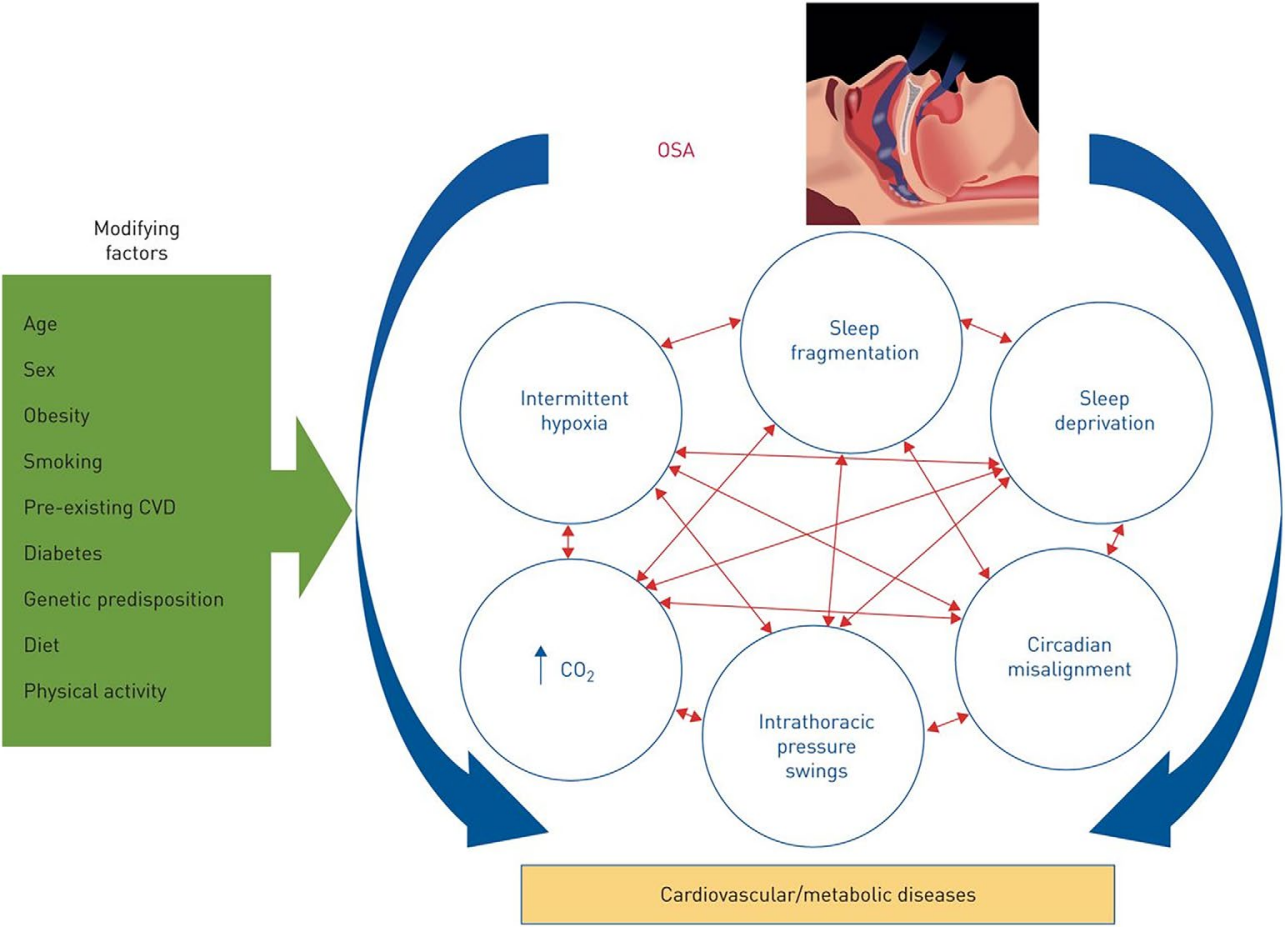
The complex pathophysiology of cardiovascular and metabolic diseases in OSA (reproduced from Ryan et al. 2020. Figure is reproduced and requires permission).

Although IH is likely the key pathogenic factor for cardiometabolic disease processes, it is for certain not the only one. In fact, OSA comprises various potential adverse triggers displaying a complex interaction (Figure 2) but the relative contributions of those factors to the actual disease processes are still unknown (Ryan 2018; Ryan et al. 2020).

Importantly, the individual susceptibility to cardiometabolic diseases in OSA subjects is substantially influenced by many modifying factors, including demographics, comorbidities, genetic influences and lifestyle (Ryan et al. 2020), and hence, there simply cannot be a ‘one size fits all’ treatment approach which is also reflected in a widely varying response to CPAP therapy in terms of cardiometabolic outcomes. Effective treatment strategies need to be personalised but also holistic taking the heterogeneity and complexity of the condition into account. Of particular importance in this regard is to target obesity which is intimately linked to OSA with at least two third of OSA subjects having a body mass index (BMI) > 30 (Peppard et al. 2013). Many subjects could likely be prevented from developing OSA if obesity would be adequately addressed in primary prevention strategies. As a treatment, weight loss is effective in reducing OSA severity with associated improvement in blood pressure, circulating inflammatory markers and insulin sensitivity which has been corroborated by several meta‐analyses (Chirinos et al. 2014; Malhotra et al. 2024a; Stelmach‐Mardas et al. 2021). Therefore, a meaningful improvement in OSA‐related adverse outcomes needs to include addressing of this important comorbidity.

## 6 Treating OSA—What Have We Learned?

As outlined above, OSA is a highly heterogeneous and complex condition with different clinical, pathophysiological, and sleep diagnostic phenotypes, and there is an increased understanding that treatment strategies should be tailored to an individual's profile and susceptibility to adverse consequences (Turnbull and Stradling 2023). The AHI is clearly insufficient to determine treatment decisions, and multicomponent systems based on phenotypic characteristics are warranted. The recently described Baveno classification incorporating symptomatology and comorbidities may be of added value in therapeutic guidance (Randerath et al. 2021a) but prospective, randomised‐controlled trials using this grading system are so far lacking.

Since its development and application to treating OSA over 40 years ago (Sullivan et al. 1981), CPAP therapy has remained the dominant modality for the majority of OSA subjects and has spawned not only an entirely new field of respiratory care and research (namely that of nocturnal ventilation) but has contributed to the establishment of sleep medicine centres worldwide to care for this ubiquitous condition, thereby helping to establish guidelines and standards for the assessment and management of the entire spectrum of sleep disorders.

However, the treatment model of CPAP delivered by an air compressor to the UA via a nasal/oronasal mask during sleep has been little bettered. New interfaces and new algorithms have been developed incrementally using improved technologies, but the principles of treatment have remained the same. This method of abolishing nocturnal obstructions of the UA irrespective of endotype or phenotype is balanced by the often‐indiscriminate application of it to patients who may not benefit in the first instance, and the potential discomfort and inconvenience of using a long‐term treatment for a condition without a cure.

Despite those drawbacks, CPAP remains a cheap, reversible, and reusable treatment fit for purpose in both public and private healthcare systems, largely straightforward in its delivery, and highly effective if offered to the appropriate patient (Table 2).

**TABLE 2:** Potential benefits and drawbacks of CPAP therapy in the treatment of OSA/OSAS.

Potential benefits	Potential drawbacks
Reasonably easy access and availability irrespective of healthcare system.	The treatment is not a cure and must be applied on an almost nightly basis for beneficial effects to be maintained.
2 Relatively low cost by comparison to other treatments	2 Cumbersomeness and inconvenience of treatment can contribute to unsatisfactory compliance.
3 Treats nocturnal sleep‐disordered breathing effectively irrespective of endotype, physiological circumstance, age, medications, allergies or co‐morbid conditions	3 The machine and paraphernalia associated with it can be awkward in certain situations, that is travel.
4 Consistent improvements in blood pressure, endothelial function, and potential other cardiometabolic benefits	4 CPAP cannot be used in situations where there is no access to a reliable power supply.
5 Favourable cost–benefit/effectiveness for individuals and society regarding treating excessive daytime sleepiness for those in vigilance‐critical professions as well as professional driving.	5 Not all personalities accept its long‐term use even if it is beneficial.
6 CPAP therapy can easily be followed through telemonitoring	6 Some components of the machines are dependent on finite resources, including rare metals.
7 The treatment is completely reversible, rarely causes harm unless injudiciously applied and has a very low side‐effect profile.	

**FIGURE 3:**
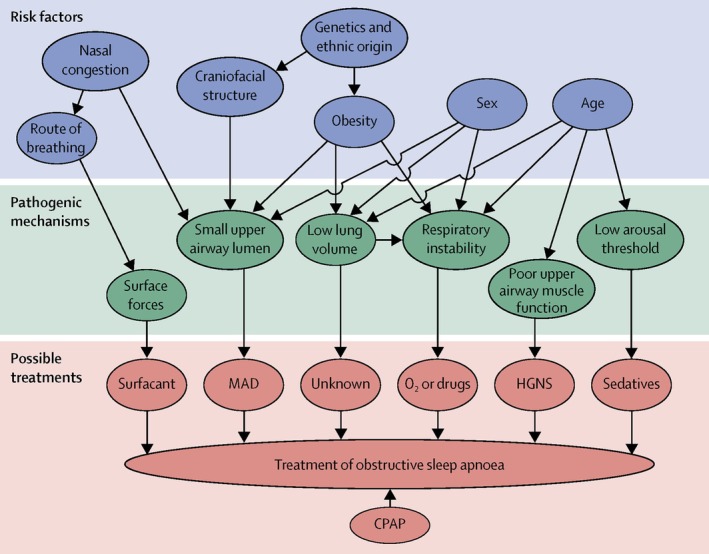
Risk factors and endotypes which might result in more personalised treatment approaches for OSA (Reproduced from: Jordan et al. 2014). Figure is reproduced and requires permission.

In general, very few alternative or newer treatments in development can compete with CPAP in effectiveness on the same scale at present, and frequently, widespread usage is also hindered by high cost and lack of availability and accessibility. Nonetheless, with the increasing understanding of different pathophysiological endotypes, especially the contribution of non‐anatomical factors, alternative management options to CPAP may play a role in selected subjects (Jordan et al. 2014) (Figure 3).

Oral appliances, such as mandibular advancement devices (MAD), are the best‐studied alternative to CPAP and are recommended for subjects who have failed or refused CPAP and also as primary therapy in patients with milder disease (Randerath et al. 2021b). The goal of therapy with oral appliances is to modify the position of the mandible in order to enlarge the airway, reduce resistance, and presumably reduce UA collapsibility (Verbraecken et al. 2022).

UA surgery is generally of greatest utility in subjects who have a well‐defined, correctable structural abnormality compromising the UA diameter. A reasonably new but not globally available treatment for OSA is hypoglossal nerve stimulation. The most commonly commercially available system involves a subcutaneously implanted device connected to a branch of the right hypoglossal nerve, which stimulates the genioglossus muscle with each inspiration, thus, counteracting UA obstruction. In selected subjects, this treatment is effective in reducing AHI; however, high cost, lack of reimbursement in many countries, and the need for specialised expertise are considerable limitations (Strollo Jr. et al. 2014).

Potential pharmacological approaches include combined noradrenergic plus antimuscarinic agents to increase dilator muscle contractility (Taranto‐Montemurro et al. 2019), carbonic anhydrase inhibition to reduce high loop gain (Edwards et al. 2012), or hypnotic agents such as zolpidem, eszopiclone, or trazodone to increase the arousal threshold (Messineo et al. 2020; Eckert et al. 2011; Eckert et al. 2014). These treatment options may have a role in selected patients where such factors are found to be contributory to the integrated pathophysiology of the disorder, but the research is still in its infancy. Currently available equipment and technology do not allow for the identification of specific OSA endotypes in routine clinical practice and thus, such treatments remain largely in the realm of research trials or highly specialised centres.

The role and suitability of treatment strategies in OSA/OSAS will ultimately be determined based on their effectiveness to mitigate daytime symptoms of impairment and potentially on the basis of cardiometabolic outcomes. Despite the negativity of recent RCT's on the cardiovascular benefits of CPAP use, it is important to understand that those studies were influenced by substantial limitations (Cohen et al. 2024; Pack et al. 2021). In contrast, analyses from large real‐world studies reveal a consistent reduction in mortality and non‐fatal CV events (Gerves‐Pinquie et al. 2022; Pepin et al. 2022; Dodds et al. 2020). Moreover, RCT's have so far focused on subjects with pre‐existing CV disease, but there is growing evidence that CPAP therapy may be of greater benefit in the early stages of cardiovascular or metabolic diseases and thus, as a form of primary prevention. In support of this viewpoint, several randomised and non‐randomised studies have demonstrated a significant benefit of CPAP therapy on endothelial dysfunction (Schwarz et al. 2015; Xu et al. 2015), blood pressure (Labarca et al. 2021; Pengo et al. 2020), insulin resistance (Jullian‐Desayes et al. 2015; Iftikhar et al. 2015) and on early vascular inflammation (Jelic et al. 2008; O'Donnell et al. 2024). Hence, CPAP therapy likely plays an important role in the management of various cardiometabolic complications in OSA, but there is considerable heterogeneity in the response, and further detailed work needs to determine how and in whom it is best deployed.

There is still a paucity of data on the cardiometabolic effects of alternative treatments in OSA. Reducing the severity of obesity may provide considerable benefit. However, weight reduction using conventional measures alone is limited in its efficacy for the majority of subjects, and bariatric surgery, the most efficient weight loss strategy, is, due to its invasive nature, not a feasible option for many OSA subjects. Pharmacotherapy with glucagon‐like peptide‐1 (GLP‐1) based medicines has expanded rapidly in recent years, and these medications are now delivering significantly better outcomes than conventional measures alone, with some dual and triple receptor agonists achieving over 20% weight loss (Jastreboff et al. 2022; Jastreboff et al. 2023). Malhotra and co‐workers recently reported data from the SURMOUNT‐OSA study demonstrating substantial reductions in OSA severity with the dual receptor agonist Tirzepatide (Malhotra et al. 2024b). In line with the change in anthropometric parameters, this was also associated with a decrease in blood pressure, especially in CPAP‐naïve subjects. How this translates into ‘hard’ cardiovascular outcomes, long‐term side effects, and adherence in the obese non‐diabetic population remains unknown. Furthermore, substantial regional differences in the availability and accessibility of GLP‐1 based pharmacotherapies, their high cost, and the lack of reimbursement schemes in many countries raise substantial concerns regarding health inequities that need to be addressed.

## 7 On Treatment and Still Sleepy—What to Do?

As discussed above, there are multiple causes of EDS and its existence in subjects with an elevated AHI/AH may be entirely coincidental. In the context of obesity, comorbid conditions, medications, lifestyle issues, circadian rhythm abnormalities, often undiagnosed psychological/psychiatric disorders, etc., a direct relationship can be particularly difficult to work out (Craig et al. 2022). One possible strategy to answer this question is to trial CPAP for a period of months to assess efficacy, although placebo effects have been observed.

The term ‘residual’ sleepiness is often used in the context of adequately treated nocturnal apnoeas/hypopnoeas, but ongoing symptoms have been poorly studied in a thorough or objective manner. A significant proportion of subjects on CPAP fall within this category (Weaver et al. 2007). It is imperative to follow a rigorous stepwise approach in determining the cause of the persistent symptoms, including optimisation of CPAP therapy and investigation for alternative causes of EDS such as additional sleep disorders (Craig et al. 2022; Barateau et al. 2024). The latter is particularly relevant for subjects with co‐morbid insomnia (COMISA), who have worse sleep, mental health, physical health, and quality of life compared to patients with either insomnia or OSA alone (Sweetman et al. 2023). CPAP usage is generally lower in COMISA than in OSA alone (Philip et al. 2018; Wallace et al. 2018) and insomnia‐targeting strategies such as cognitive behavioural therapy for insomnia (CBT‐I) may be beneficial (Alessi et al. 2021; Sweetman et al. 2019). For those where no obvious cause of the residual sleepiness can be found, a number of wake‐promoting agents have been shown to improve EDS (Pepin et al. 2021; Schweitzer et al. 2019) and have subsequently been approved in some countries. However, the use of stimulant medication to treat ‘residual sleepiness’ has not been studied over the long term to ascertain the degree of harm or benefit. Although much time, energy, and money have gone into assessing directly the effects of IH and arousals at a cellular and physiological level, very little research has been expended over the decades on measuring and assessing sleepiness, tiredness, and fatigue per se, which remains an important shortfall.

## 8 Conclusion

Though by no means exhaustively, we have attempted to raise and address a number of outstanding issues regarding the diagnosis, pathophysiology, and treatment regarding OSA and OSAS as they currently stand. CPAP is unlikely to disappear from the armamentarium for treating sleep disordered breathing at night and will continue to benefit large swathes of the population suffering from this common disorder. However, future management of OSA and OSAS lies in a more personalised and holistic approach informed by a more precise definition of OSA, a better understanding of the clinical, pathophysiological, sleep diagnostic, and susceptibility phenotypes, as well as the role of comorbidities.

## Author Contributions

**Maria Stanczyk:** writing – original draft, conceptualization, writing – review and editing. **Walter T. McNicholas:** conceptualization, writing – original draft, writing – review and editing. **Dirk A. Pevernagie:** conceptualization, writing – original draft, writing – review and editing. **Renata L. Riha:** conceptualization, writing – original draft, writing – review and editing. **Silke Ryan:** conceptualization, writing – original draft, writing – review and editing.

## Conflicts of Interest

The authors declare no conflicts of interest.

## Data Availability

Data sharing not applicable to this article as no datasets were generated or analysed during the current study.
